# Validation and Cross-cultural Adaptation of the Sinonasal Outcome Test (SNOT)-22 for the Arabian Patient Population

**DOI:** 10.7759/cureus.4447

**Published:** 2019-04-12

**Authors:** Mohammed Asiri, Ghassan Alokby

**Affiliations:** 1 Otolaryngology, King Abdulaziz Medical City, Ministry of National Guard Health Affairs, Riyadh, SAU; 2 Otolaryngology, Prince Sultan Military Medical City, Riyadh, SAU

**Keywords:** chronic rhinosinusitis, quality of life, arabic, sinus surgery, snot-22, sinonasal outcome test, sinusitis, validation, snot, crs

## Abstract

Background and objective

Quality of life measurement is an essential element of healthcare evaluation. The Sino-Nasal Outcome Test 22 (SNOT-22) has been validated in different languages, and in this study we validated the SNOT-22 in Arabic language. The objective of this study is to provide a validated, cross-culturally adapted version of the SNOT-22 for the Arabic speaking population.

Materials, methods, and main outcome measures

This was a prospective cohort study set in a tertiary hospital in Riyadh, Saudi Arabia. The SNOT-22 was translated into Arabic by two native Arabic speakers. A total of 30 patients with chronic rhinosinusitis (CRS) with/without nasal polyps were included in test-retest study. Following that, a prospective study was conducted where the translated SNOT-22 was distributed to a different set of 30 CRS patients before and three months after endoscopic sinus surgery. Another 50 healthy individuals were included as a control group. The main outcome measure was the translation and validation of the SNOT-22 in Arabic.

Results

Internal consistency was assessed by performing a test-retest study. Cronbach’s alpha was 0.803 at both the initial examination and at the retest, showing good internal consistency. There was a statistically significant difference between the results of the control group and the preoperative results of the CRS group (p<.001). The preoperative mean (SD) SNOT-22 score for the CRS group was 64.8 (20.3) and it dropped to 29.2 (11.8) postoperatively showing statistically significant change (p<.001), indicating the responsiveness of the SNOT-22.

Conclusion

The Arabic version of the SNOT-22 has internal consistency, reliability, and reproducibility that is needed for it to be a valid instrument to be used in research and clinical practice.

## Introduction

Chronic rhinosinusitis (CRS) is a well-known disease that causes inflammation of the nasal sinuses and the linings of the nasal passages. In 2012, the European Position Paper on Rhinosinusitis and Nasal Polyps (EPOS) proposed diagnostic criteria for CRS, which includes the following: two or more symptoms, one of which should be either nasal blockage or nasal discharge, ± facial pain/pressure, ± hyposmia or anosmia; and either endoscopic signs of polyps and/or mucopurulent discharge from middle meatus and/ or edema/mucosal obstruction in middle meatus and/or CT findings showing mucosal changes within the ostiomeatal complex and/or sinuses. The duration of symptoms must be for 12 weeks or more [1].

CRS affects 14%-15% of the adult United States (US) population, while in Europe it has been reported to affect 5%-15% of the population [2]. Because CRS has been shown to substantially reduce the health-related quality of life of the affected patients, the assessment of quality of life (QoL) becomes an essential element of healthcare evaluation, and the burden and relief of symptoms as perceived by the patient plays an essential role in the choice and evaluation of treatment by the clinician [3].

As CRS causes significant impact on patients’ quality of life, many disease-specific QoL instruments were developed, such as the Sinonasal Outcome Test (SNOT), the Sino-Nasal Outcome Test 20 (SNOT-20) and 22 (SNOT-22), which are validated patient-reported measures of symptom severity and health-related QoL in sinonasal conditions [2, 4].

SNOT-22 is the modified version of SNOT-20, to which two items have been added-nasal blockage and sense of taste and smell [2]. SNOT-22 covers a broad range of health-related QoL problems including physical problems, functional limitations, and emotional consequences [5]. The outcomes it measures can be divided into four different clinical subscales: rhinological symptoms (items 1-7, 12), ear and facial symptoms (items 8-11), sleep function (items 13-16), and psychological issues (items 17-22).

The SNOT-22 questionnaire was initially written in English, but it has been translated to and validated in different languages [2, 6-14]. To the best of our knowledge, this is the first translation of the Arabic SNOT-22 that has been tested for validity and responsiveness by comparing the score of CRS patients to a control group of healthy individuals and by comparing the score of CRS patients before and after surgical intervention.

## Materials and methods

**Figure 1:**
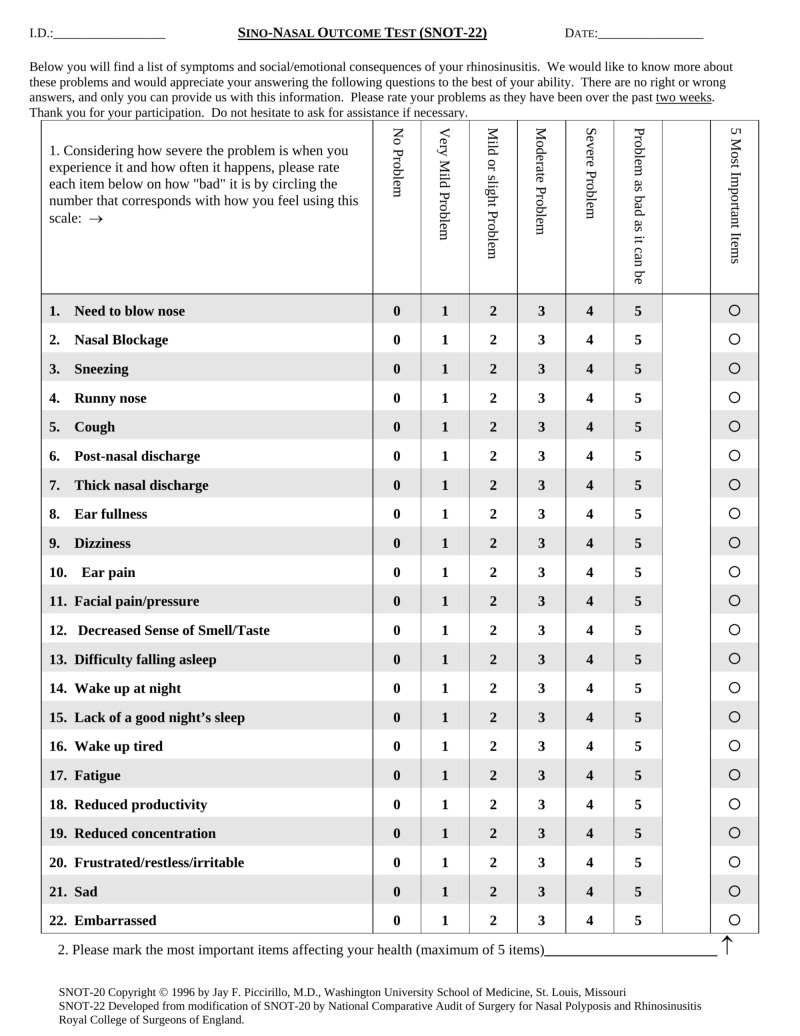
The SNOT-22 in English

**Figure 2:**
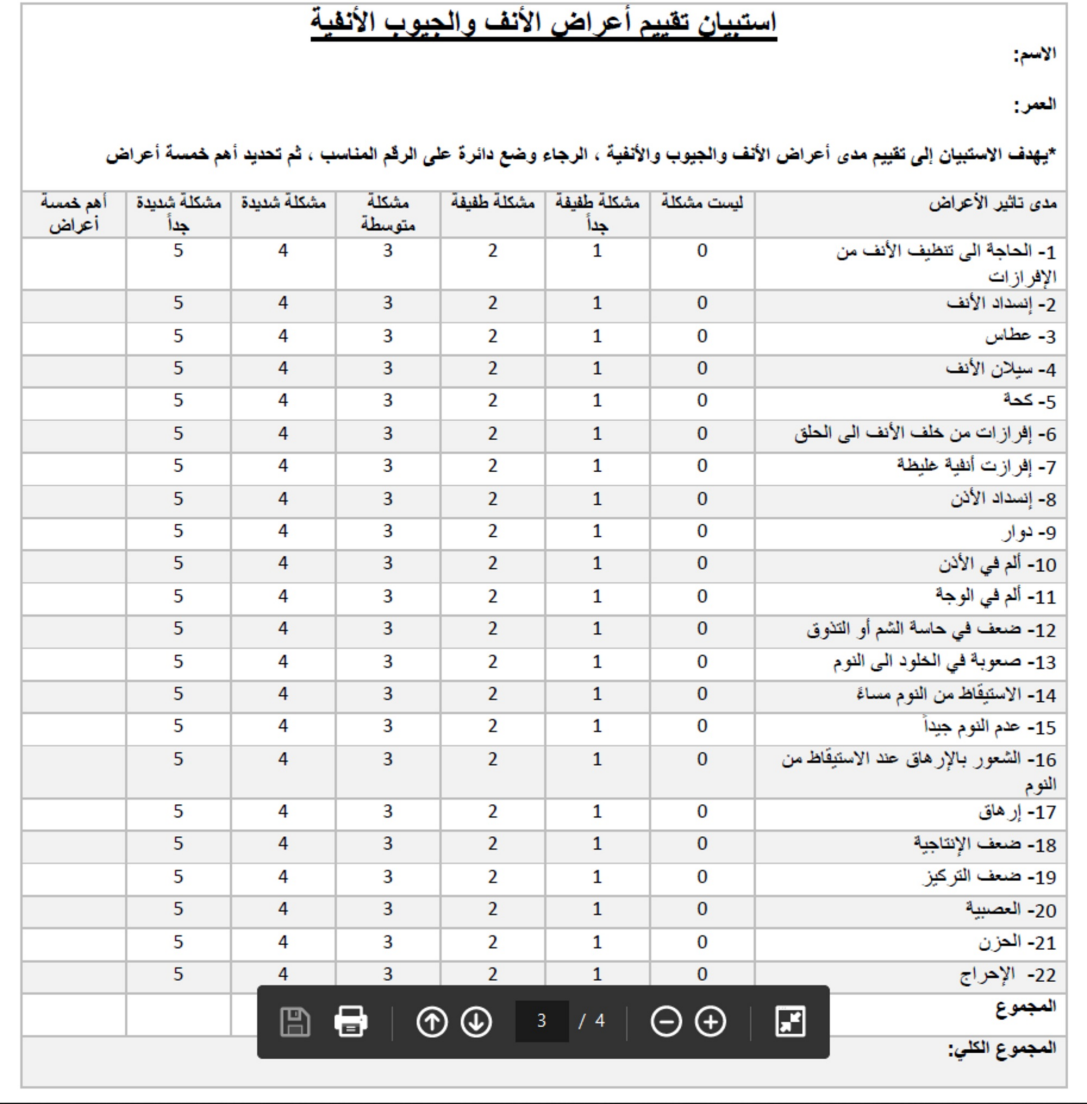
Arabic version of the SNOT-22

Initially, two independent native Arabic translators translated the original SNOT-22 questionnaire from English to Arabic language, after which two other native English translators re-translated it from Arabic to English. The English and Arabic versions of SNOT-22 are shown in Figures 1-2.

The inclusion criteria were adult patients who fit the diagnostic criteria of EPOS for CRS (with/without nasal polyps). The exclusion criteria were age less than 18 years, pregnancy, and refusal to participate in the study. The test-retest reliability was calculated by getting patients with CRS to fill the SNOT-22 questionnaire in two different clinic visits by two different physicians.

A control study was done and the control group consisted of members of the hospital employees and accompanying persons or relatives. We excluded any participant who had a history of CRS and/or using nasal medication. All subjects underwent nasal endoscopic examination first and then SNOT-22 scores were obtained.

A preoperative-postoperative study was also conducted. Patients undergoing sinus surgery were evaluated using the Arabic version of the SNOT-22 questionnaire one day before surgery and three months after.

Statistical analysis was done using the Statistical Package for Social Sciences (SPSS, IBM Corp., Armonk, USA) software and the level of significance was (p<.05). The internal consistency of the Arabic version of SNOT-22, which reflects the way how the items relate to each other was analyzed; Cronbach’s alpha was used to represent and evaluate internal consistency for ordinal responses. The minimum acceptable value was 0.7. Cronbach’s alpha coefficient was calculated for each item and by removing each item at once after.

Using Pearson’s test and kappa test (reproducibility) the test-retest reliability, which indicates stability over time when repeating the test, was analyzed by correlating the initial test with the subsequent scores. The responsiveness of the Arabic version of SNOT-22 was assessed by comparing the SNOT scores before and after surgery. The paired t test was used for the statistical evaluation. The Wilcoxon signed-rank sum test (Z) was performed when normality of the differences was not fulfilled. Spearman's correlation and Pearson’s coefficient (R) were used for analysis of ordinal or non-parametric variables.

## Results

A total of 30 patients with CRS with/without nasal polyps were included in test-retest study. Of the subjects, 36.7% were females (n=11) and 63.3% were males (n=19). The mean (SD) SNOT-22 sum score was 64.2 (7.9) (range: 52 to 85) in the initial test, and 65.1 (7.8) (range: 51 to 84) in the retest. The Wilcoxon signed-rank sum result for the test was 1.815 (p=.069), (Table 1).

**Table 1:** Total SNOT-22 scores of the groups in validation study

Group	n	Mean ± SD	Range
Initial test	30	64.2 (7.9)	52 – 85
Retest	30	65.1 (7.8)	51 – 84
Control	50	19.5 (13.1)	0 - 57
Preoperative	30	64.8 (20.3)	25 - 104
Postoperative	30	29.2 (11.8)	6 - 53

The Cronbach’s alpha result of the initial examination was 0.803 and 0.803 for the retest examination, which suggests good internal consistency with the Arabic SNOT-22.

Pearson’s correlation analysis was calculated with an R value of 0.907 (p<.001) and kappa of 0.161, which indicates a strong correlation between the initial test and the retest examination scores. The results of Cronbach’s alpha and Spearman's correlation for each item in the test and retest examinations are given in Table 2.

**Table 2:** Cronbach’s alpha and Spearman's correlation results for each item

SNOT-22 Item	Item description	Cronbach’s alpha		Spearman's correlation	
		Test	Retest	Test	Retest
1	Need to blow nose	0.806	0.806	0.941	0.925
2	Nasal obstruction	0.805	0.805	0.912	0.867
3	Sneezing	0.801	0.805	0.857	0.834
4	Runny nose	0.805	0.804	0.843	0.886
5	Cough	0.804	0.804	0.958	0.962
6	Post nasal discharge	0.804	0.804	0.959	0.968
7	Thick nasal discharge	0.805	0.806	0.956	0.909
8	Ear fullness	0.809	0.809	0.963	0.960
9	Dizziness	0.806	0.806	0.935	0.949
10	Ear pain	0.805	0.805	0.920	0.905
11	Facial pain/pressure	0.797	0.799	0.936	0.885
12	Loss of smell or taste	0.804	0.803	0.867	0.866
13	Difficulty falling asleep	0.800	0.801	0.949	0.957
14	Waking up at night	0.802	0.801	0.965	0.955
15	Lack of a good night’s sleep	0.795	0.796	0.964	0.948
16	Waking up tired	0.794	0.794	0.966	0.952
17	Fatigue	0.807	0.805	0.853	0.851
18	Reduced productivity	0.798	0.799	0.988	0.975
19	Reduced concentration	0.798	0.797	0.942	0.883
20	Frustrated/restless/irritable	0.803	0.802	0.957	0.959
21	Sad	0.806	0.807	0.951	0.942
22	Embarrassed	0.806	0.805	0.981	0.985

The control group in the control study included 50 volunteers from hospital employees and accompanying persons or relatives of our patients. Of the subjects 14.0% were females (n=7) and 86.0% were males (n=43). The mean (SD) total score for the control group was 19.5 (13.1).

The mean (SD) total SNOT-22 score was significantly higher with the group of patients before surgery (64.8 (20.3)) compared to the mean (SD) total SNOT-22 score of the control group (19.5 (13.1)), (p<.001), which indicated a statistically significant difference between the two groups.

Thirty patients were included in the preoperative-postoperative study, and 36.7% of them were females (n=11) and 63.3% were males (n=19). The mean (SD) preoperative SNOT-22 total score was 64.8 (20.3) (range: 25 to 104) and the mean (SD) postoperative score was 29.2 (11.8) (range: 6 to 53). The mean score after three months postoperatively was significantly lower (p<.001), indicating the responsiveness of the SNOT-22 (Table 1).

## Discussion

There are over 15 known disease-specific sinonasal outcome questionnaires in English [12]. Among them is the SNOT-22 questionnaire, which showed reliability, validity, responsiveness, and ease of use [15]. This has made it widely used both in research and in clinical practice for patients with CRS. The SNOT-22 is already validated in many languages including Czech, Danish, Greek, Lithuanian, Persian, and Spanish [2, 6, 9, 11-14].

Following the generally accepted methodology as described by Koller et al., the translation, cross-cultural adaptation, and validation of the Arabic version of the SNOT-22 questionnaire was carried out [16]. Internal consistency score refers to the relation of items within an instrument. The minimum acceptable value for Cronbach’s alpha test is considered to be 0.7; above 0.8 is considered good and more than 0.9 is considered excellent.

Cronbach’s alpha was calculated for the initial test and the retest with a value of 0.803, which indicates a good internal consistency. Schalek et al., Lange et al., and Vaitkus et al. evaluated Pearson’s test in SNOT-22 [2, 6, 9]. They reported a Pearson’s correlation coefficient of 0.86, 0.70 and 0.72, respectively. In our study, Pearson’s correlation coefficient R value was 0.907 (p<.001), which suggests a strong correlation between the initial test and the retest scores.

The evaluation of reproducibility was done using kappa test with a score of 0.161.

We tested the ability of the Arabic version of SNOT-22 to reflect the differences between CRS patients groups, which consist of 30 patients with a control group of 50 healthy individuals, by comparing the mean SNOT-22 scores for each group. The mean (SD) SNOT-22 scores of patients with CRS was 64.8 (20.3) compared to the healthy individuals' mean (SD) of 19.5 (13.1), indicating a statistically significant difference between the two groups (p<.001).

Responsiveness is defined as the ability of a questionnaire to detect changes over time. The responsiveness of the SNOT-22 questionnaire was assessed by comparing the final scores before and after the surgical intervention (sinus surgery) using the paired t test. The paired t test showed that SNOT-22 scores pre- and postoperatively were statistically significant (p<.001). The mean (SD) preoperative SNOT-22 total scores were 64.8 (20.3) (range: 25 to 104) and the mean (SD) postoperative total scores was 29.2 (11.8) (range: 6 to 53).

Three months postoperatively, the mean SNOT-22 score was significantly lower than that of the preoperative examination (p<.001), which indicates an excellent responsiveness to SNOT-22.

## Conclusions

In conclusion this study proves that the Arabic version of the SNOT-22 questionnaire has the internal consistency, reliability, and reproducibility that is needed for it to be a valid instrument to be used in research and clinical practice.
